# Impact of the COVID-19 Pandemic on Elective and Emergency Surgical Procedures in a University Hospital

**DOI:** 10.1590/0100-6991e-20223324-en

**Published:** 2022-08-17

**Authors:** MATEUS ROCCO, BRUNA LARISSA DE OLIVEIRA, DARINA ANDRADE ADDARIO RIZZARDI, GABRIEL RODRIGUES, GABRIELY DE OLIVEIRA, MILENA GONÇALVES GUERREIRO, VINÍCIUS SIPPEL CRUZ, CARLOS ROBERTO NAUFEL-JUNIOR

**Affiliations:** 1 - Faculdade Evangélica Mackenzie do Paraná - Curitiba - PR - Brasil; 2 - Centro Universitário da Fundação Assis Gurgacz - Cascavel - PR - Brasil; 3 -Centro Universitário São Camilo - São Paulo - SP - Brasil; 4 - Universidade Federal do Mato Grosso - Sinop - MT - Brasil; 5 - Universidade de Araraquara - Araraquara - SP - Brasil

**Keywords:** COVID-19, Elective Surgical Procedures, Pandemics, Hernia, Cholecystectomy, COVID-19, Procedimentos Cirúrgicos Eletivos, Pandemias, Hérnia, Colecistectomia

## Abstract

**Objective:**

to assess the impact of the COVID-19 pandemic on abdominal wall hernia repair surgeries and cholecystectomy in a referral center hospital.

**Methods:**

a retrospective, observational, cross-sectional study carried out at Hospital Universitário Evangélico Mackenzie (HUEM), in Curitiba, Paraná, Brazil. Data obtained through electronic medical records of patients who underwent cholecystectomy and abdominal wall hernia repair from March to December 2019 and 2020 at HUEM were included. Data were analyzed using Pearsons Chi-Square test and analysis of variance (ANOVA).

**Results:**

a total of 743 medical records were analyzed, with a 63.16% drop in the total number of surgeries in 2020. There was a 91.67% increase in the number of ICU admissions in 2020, as well as a 70% increase in average length of stay. A greater number of complications was observed (in 2020, 27% had complications, while in 2019 this figure was 18.8%) and an increase in mortality (in 2019, this rate was 1.3% and in 2020, 6.5%). There were 6 cases of COVID-19 in 2020, so that of these, 5 patients died.

**Conclusion:**

during the COVID-19 pandemic, an important reduction in the number of abdominal wall hernia repair surgeries and cholecystectomy was observed. In addition, there was a statistically significant increase in postoperative complications, mortality rate and length of stay in 2020.

## RESUMO

**Objetivo:**

avaliar o impacto da pandemia da COVID-19 em cirurgias de reparo de hérnias de parede abdominal e colecistectomia em hospital centro de referência.

**Métodos:**

estudo transversal retrospectivo observacional realizado no Hospital Universitário Evangélico Mackenzie (HUEM), em Curitiba, Paraná, Brasil. Foram incluídos os dados obtidos através de prontuários eletrônicos de pacientes que realizaram colecistectomia e reparo de hérnias de parede abdominal no período de março a dezembro de 2019 e 2020 no HUEM. Os dados foram analisados por meio do teste Qui-Quadrado de Pearson e aplicação da Análise de Variância (ANOVA).

**Resultados:**

Foram analisados 743 prontuários ao todo, sendo constatada uma queda de 63,16% no número total de cirurgias no ano de 2020. Verificou-se um aumento de 91,67% no número de internações em UTI em 2020, bem como um aumento de 70% no tempo médio de internação. Foi observado um maior número de complicações (em 2020, 27% apresentaram complicações, enquanto em 2019 este valor foi de 18,8%) e um aumento em relação à mortalidade (em 2019, esta taxa foi de 1,3% e em 2020, 6,5%). Observaram-se 6 casos de COVID-19 em 2020, de modo que destes, 5 pacientes vieram a óbito.

**Conclusão:**

durante a pandemia da COVID-19, observou-se uma importante redução na quantidade de cirurgias de reparo de hérnia de parede abdominal e colecistectomia. Além disso, houve aumento estatisticamente relevante quanto às complicações pós-operatórias, taxa de mortalidade e tempo de internamento em 2020.

## INTRODUCTION

In January 2020, the World Health Organization (WHO) issued a global health alert for a novel coronavirus called severe acute respiratory syndrome coronavirus 2 (SARS-CoV-2), which originated in Wuhan, Hubei Province, China. The COVID-19 pandemic, declared by the WHO in March 2020, resulted in more than 770,000 cases worldwide reported by the end of March 2020^1^. By November 2021, due to its high transmissibility, more than 45,000,0000 cases had been confirmed across 219 countries^2^.

As the need for hospitalization among symptomatic cases is 10% globally, with an increase in the need for admission to an intensive care unit and 3% mortality, hospitals started to intensively reduce elective activities, including surgeries, to be prepared for the high number of admissions. Therefore, the COVID-19 pandemic caused major disruptions in routine hospital services around the world^3^ ^-^ ^5^.

Among the main services interrupted were elective surgeries, which were canceled to offer greater safety to the patients. Reducing elective activities protects patients from in-hospital transmission of the virus and associated postoperative pulmonary complications^3^ ^,^ ^5^. In addition, protective supplies are preserved, which are prioritized for the care of patients with COVID-19^3^ ^,^ ^4^. Furthermore, the surgical team can be reassigned to other specialties and to the front line, if necessary^6^. It is estimated that 72.3% of surgeries were canceled during the peak of the pandemic, in its first 12 weeks, around the world^7^.

Given that approximately six million procedures are performed weekly around the world, the cancellation of elective surgeries during the pandemic would soon generate an accumulation^9^. The impacts of this reality can be seen in the most diverse areas, causing economic impacts and an increase in morbidity and mortality for patients who had their surgeries postponed^10^.

Among the elective surgical procedures most performed in hospital centers are cholecystectomy and hernia repair. The first consists of removing the gallbladder, the laparoscopic approach being the most used today. It is one of the most performed abdominal surgical procedures in the US every year^11^. Hernia repair, on the other hand, mainly involves the closure of the abdominal wall defect through which the organ protruded, performed through a tension-free repair, the mesh one being usually chosen for this^12^.

Studies sought to assess the impact of the COVID-19 pandemic on both procedures, evaluating aspects such as the volume of surgeries during the pandemic, the incidence of operative complications, and even changes in the approach to these procedures to bring more safety to the patient. The present work seeks to better understand the impact of the pandemic on cholecystectomy and hernia repair surgeries at the Hospital Universitário Evangélico Mackenzie (HUEM), a University Hospital in Curitiba, State of Paraná, Brazil.

## METHODOLOGY

### Study design

Retrospective, observational, cross-sectional study.

### Location

The research was carried out at Hospital Universitário Evangélico Mackenzie (HUEM), in the city of Curitiba, Paraná, Brazil.

### Research subjects

We included patients over 18 years of age who underwent elective cholecystectomy and abdominal wall hernia repair from March to December 2019 and March to December 2020. We included the ICD (International Statistical Classification of Diseases and Related Health Problems) codes K40, K41, K42, and K43 for abdominal wall hernias, and K80 and K81 for cholelithiasis and acute calculous cholecystitis.

### Data collection

We carried out the data collection in February 2021, through analysis of electronic medical records.

### Data analysis

The variables compared in the medical records of patients who underwent elective, open or laparoscopy cholecystectomy surgery and elective abdominal wall hernia repair surgery were age, sex, length of stay, number of infected with COVID-19 during the hospitalization period, postoperative complications, intraoperative complications, delay in seeking medical care, and mortality rate. To verify the significance of the relationship between the findings, we applied parametric statistical tests (Pearson’s Chi-square, Application of Analysis of Variance - ANOVA) through the SPSS Software - Statistical Package for the Social Sciences -, adopting a confidence interval of 95% and 5% error margin (p-value <0.05).

### Literature review

We carried out a literature review with searches in the PubMed database using the following descriptors: “COVID and hernia”, “COVID and cholecystectomy”, “cholecystectomy”, “hernia repairs”, “surgery and COVID”, “surgery and hernia and “COVID”, “impact and surgery and COVID” and “epidemiology and surgery and COVID”. We included articles that addressed the objectives of the present study, without restriction as to their publication date.

### Ethical issues

We performed the research with prior written authorization signed by the person responsible for the research institution and approval by Plataforma Brasil. Only the researchers had access to the collected data, which were kept in a safe and confidential place. The project was submitted to the Committee of Ethics in Research with Human Beings of the Faculdade Evangélica Mackenzie do Paraná, as established by law, with Certificate of Presentation for Ethical Appreciation - CAAE - number 42959121.7.0000.0103

## RESULTS

**Figure 1:**
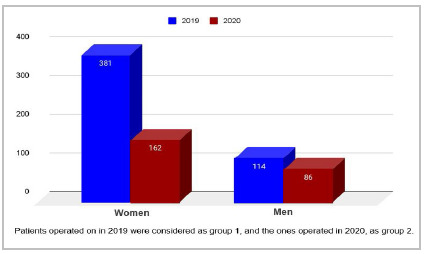
Patients in 2019 versus 2020.

The study included 743 medical records of patients who underwent abdominal wall hernia repair and cholecystectomy in the period. The 2019 patients were considered as group 1, and the 2020 ones, as group 2, as shown in Figure 1. Of the surgeries performed in 2019 and 2020, 543 occurred from March to December 2019 and 200 occurred in the same period of 2020, which represents a decrease of 63.16% in the volume of surgical procedures. In 2019, there were 348 cholecystectomies and 209 abdominal wall hernia repairs. In 2020, there were 148 cholecystectomies and 103 hernia repairs. There were 381 women and 162 men in 2019, and 114 women and 86 men in 2020. In 2019, 286 patients had some previous comorbidity, the same being true for 105 patients in 2020. The age range was similar between the two groups, group 1 having a mean ± standard deviation of 46.92 ± 15.18, and group 2, 48.42 ± 15.57.

The quantitative variables analyzed were age group, morbidity, mortality, and the volume of surgical procedures performed. Statistical analysis found no significant difference (p>0.05) between the mean age. However, we observed a statistically significant difference (p<0.05) in the variables morbidity, mortality, and volume of surgical procedures performed.

Regarding complications, 102 (18.8%) evolved with morbidity in group 1 and 54 (27%) in group 2, the main complications being seroma, surgical wound infection, and incisional hernia. As for mortality, seven (1.3%) patients died in group 1, and 13 (6.5%) in group 2. Among the causes of mortality, hemorrhagic shock and septic shock stand out in group 1, each type of shock being responsible for three deaths, and one death due to metabolic acidosis/hydroelectrolytic disorder. In group 2, septic shock and COVID-19 infection were the main causes, each responsible for five deaths, followed by hemorrhagic shock, associated with three deaths. Patients with comorbidities had a higher mortality rate in 2020, of 10.6%, compared with 2.4% in 2019 (p<0.05). Patients who had postoperative complications in 2020 also had a higher mortality rate, 22.2%, compared with 5.9% in 2019 (p<0.05), as shown in Table 1.

**Table 1:** Prevalence of morbidity and mortality in patients who underwent elective hernia repair and/or cholecystectomy and volume of surgical procedures in the year 2019 (March-December) and 2020 (March-December), in a University Hospital - Curitiba/PR.

	Group 1 n=543	Group 2 n=200	Total	p
Complications	102 (18,8%)	54 (27%)	158	S
Mortality	7 (1,3%)	13 (6,5%)	20	S
Volume of procedures	543	200	743	S

Group 1: patients in 2019; Group 2: patients in 2020; s: significant (p<0.5).

There was also an increase in admissions to the intensive care unit, with 12 admissions in group 1 and 23 in group 2. Regarding the length of stay, group 1 had a mean ± standard deviation of 3.51 ± 2.9, and group 2, 5.97 ± 6.9. As for the average time of seeking medical care, there was a decrease from 377.43 days in group 1 to 150.93 days in group 2.

There were six cases of COVID-19 among the 200 patients analyzed in 2020, representing 3% of patients. Of these, five (83.33%) died. The confirmation of COVID-19 infection was carried out by PCR-RT molecular test in the hospital after the surgical procedures.

## DISCUSSION

The COVID-19 pandemic has significantly impacted not only the volume of surgical procedures but also several related epidemiological aspects.

In the present study, we observed a drop of 63.16% in the total number of cholecystectomies and abdominal wall hernia repairs during the pandemic. This important reduction agrees with data found in the literature. McBride et al. found a 26% decline in elective and emergency surgical procedures when comparing the periods from February to September of 2019 and 2020 in an Australian reference center^13^. Presl et al. found a 42.5% decrease in emergency surgical procedures in Austria, also comparing the years 2019 and 2020, so that repairs of abdominal wall hernias and emergency cholecystectomies were reduced in 70% and 39%, respectively^14^.

Among the 200 patients analyzed from March to December 2020, six (3%) were diagnosed with COVID-19. Of those, five (83.33%) died after confirmation of the virus infection. In addition, only one of these patients was diagnosed with COVID-19 in the same hospitalization of the surgical procedure. The remaining patients were discharged from hospital after surgery and returned to the hospital soon after with symptoms suggestive of COVID-19 infection, the PCR-RT molecular test then having been performed at the hospital for diagnostic confirmation, which suggests that these patients came into contact with the virus during the first hospitalization but did not present clinical manifestations before discharge.

Morbidity and mortality rates showed a statistically significant increase during the pandemic period, which agrees with the literature. Cano-Valderrama et al. observed a statistically significant increase in the rate of postoperative complications when comparing groups of patients operated on before and during the pandemic, this rate rising from 34.74% to 47.01%, respectively^8^. However, the mortality rate was very similar in both groups, not representing a significant increase. Surek et al. found a statistically significant increase in the mortality rate during the pandemic, but the complication rates were similar^15^. It is also interesting to highlight that the presence of comorbidities significantly contributed to the increase in the mortality rate in 2020, with mortality rate in patients with comorbidities of 10.6%, compared with 2.4% in 2019.

We observed an important decrease in the average time for seeking medical care, which was reduced from 377.43 days in 2019 to 150.93 days in 2020. This reduction is an unexpected finding, since the literature generally reports an increase in the average time to seek care during the pandemic. Patriti et al. applied a questionnaire to surgeons in general surgery services in Italy, 40% of them reporting a significant delay on the part of patients in seeking care during the pandemic^16^. Cano-Valderrama and Surek also pointed to an increase in the time of seeking medical care during the pandemic, which has led to an increase in the number of patients with more severe clinical conditions and, therefore, to higher morbidity and mortality rates observed in these studies^8^ ^,^ ^15^.

The reduction in the average search time observed here can be attributed mainly to the prioritization of emergency cases over elective ones, whose surgeries were mostly postponed. In addition, one can assume that the higher proportion of urgent cases during the pandemic led to an increase in complication and mortality rates, as those were cases with a more severe clinical picture in general. This increase in the care of urgent cases was also observed by Patriti, showing that 79.3% of the surgeons reported having operated only on urgent cases during the pandemic period^16^.

There was also a statistically significant increase in the average length of stay in the year 2020. Some studies in the literature provide divergent data, though. Presl et al. found that the average length of stay dropped from four to three days in the pandemic, a 25% decrease^14^. The works by Patel et al. and Cano-Valderrama et al. also showed important decreases in this value^8^ ^,^ ^17^. These reductions reported in the literature are probably associated, according to the authors, with the management and rapid release of patients without COVID-19 to avoid as much as possible the contagion of these patients with the virus and to prepare the health systems for the pandemic, a strategy adopted by several countries around the world^8^ ^,^ ^14^. On the other hand, Fouad et al. evaluated the impact of the pandemic on the management of acute cholecystitis, having found an increase in the average length of stay of patients undergoing cholecystectomy in 2020 (13.5 days) compared with 2019 (2.6 days)^18^. The authors attribute these results again to the postponement of a large volume of surgical procedures, which led to the worsening of cholecystitis and, therefore, to surgeries with higher complication rates, more intraoperative complications, and, consequently, a longer hospital stay^18^.

In the present study, the increase in the average length of stay can be explained by the significant increases in morbidity and mortality rates and in the number of ICU admissions during the pandemic period (evidencing the increase in the incidence of postoperative complications). This increase may also be associated with patients infected with COVID-19 during hospitalization, who had a high average hospital stay of 29.33 days.

Our work has certain limitations, such as having been carried out in a single hospital, the scarce literature on the subject, and the failure to monitor the repercussions generated by the 2020 decisions in the year 2021, such as the possible accumulation of procedures due to the postponement of elective surgeries and its consequent increase in complications. We hope that more studies will be carried out to assess the impacts of the pandemic over the years, so that better decisions can be made in the future, considering the mistakes made in the past.

## CONCLUSION

The COVID-19 pandemic has significantly impacted hospitals around the world, especially the surgical sectors. Among the surgeries, the present study focused on hernia repairs and cholecystectomies, which sustained a reduction of more than 63% from 2019 (non-pandemic year) to 2020 (pandemic year). Due to the cancellation of elective procedures and the relocation of medical teams to the COVID-19 care sectors, non-urgent surgeries were left for a second moment, so that only urgent procedures were performed during the period. This fact was demonstrated by the decrease in the average time of seeking medical care by 226.5 days.

In addition, COVID directly impacted patients undergoing surgical procedures, since more than 80% of those affected by the virus died. There were also increases in complication and mortality rates, in the number of ICU admissions, and in the average length of stay during the pandemic. There was no significant difference in the rates of intraoperative complications.

Due to the purpose of comparing the pre-pandemic with the pandemic moment, we analyzed a heterogeneous group of patients, since there was a quantitative reduction of surgeries performed. Thus, it will be possible to continue the present study with the same methods, comparing years compromised by the pandemic, since, in this case, the infection by COVID-19 would be distributed equally among the groups analyzed.
